# Porcine Myeloid Antimicrobial Peptides: A Review of the Activity and Latest Advances

**DOI:** 10.3389/fvets.2021.664139

**Published:** 2021-05-14

**Authors:** Shuaibing Shi, Tengfei Shen, Yongqing Liu, Liangliang Chen, Chen Wang, Chengshui Liao

**Affiliations:** ^1^The Key Lab of Veterinary Biological Products, Henan University of Science and Technology, Luoyang, China; ^2^College of Animal Science and Technology/Luoyang Key Laboratory of Live Carrier Biomaterial and Animal Disease Prevention and Control, Henan University of Science and Technology, Luoyang, China

**Keywords:** antimicrobial peptide, porcine, PMAPs, bacterial resistance, biological activity

## Abstract

Traditional antibiotics have made great contributions to human health and animal husbandry since the discovery of penicillin in 1928, but bacterial resistance and drug residues are growing threats to global public health due to the long-term uncontrolled application of antibiotics. There is a critical need to develop new antimicrobial drugs to replace antibiotics. Antimicrobial peptides (AMPs) are distributed in all kingdoms of life, presenting activity against pathogens as well as anticancer, anti-inflammatory, and immunomodulatory activities; consequently, they have prospects as new potential alternatives to antibiotics. Porcine myeloid antimicrobial peptides (PMAPs), the porcine cathelicidin family of AMPs, have been reported in the literature in recent years. PMAPs have become an important research topic due to their strong antibacterial activity. This review focuses on the universal trends in the biochemical parameters, structural characteristics and biological activities of PMAPs.

## Introduction

Since the discovery of antibiotics in the twentieth century, they have been widely used in human medicine and animal husbandry due to their antibacterial, antiviral, antitumour, and growth-promoting properties (1, 2). However, with the misuse of antibiotics, bacterial resistance, antibiotic pollution, and drug residues have become global public health problems (1–3). In the UK, approximately half of deaths due to Gram-negative bacterial infection are attributed to the development of bacterial resistance (4). If the problem of antimicrobial resistance is not addressed, some common bacterial infectious diseases can threaten people's lives again (5). A total of 23,000 people die each year from antimicrobial-resistant pathogens, and the number will increase to 10 million by 2050 (6, 7). Gross domestic product will decline by ~2–3.5% per year (8), and the economic loss caused by bacterial drug resistance may reach US $2.9 trillion per year (9). Because the development of new antimicrobials has always been slower than the evolution of drug-resistant bacteria (4), we urgently need to research a new antimicrobial drug to replace antibiotics.

## Antimicrobial Peptides

Antimicrobial peptides (AMPs) are effector molecules of the innate immune defense system of animals and plants (10). AMPs have attracted extensive attention from scientists due to their low molecular weight (generally 30–60 amino acids), broad antimicrobial spectrum, and lack of induction of drug resistance (7). AMPs selectively kill bacteria, fungi, viruses, and even tumors (9), and they are a potential substitute for traditional antibiotics and are expected to be new antimicrobial drugs used in clinical applications (11). During the 1980s, the first cecropins were isolated by Hans Boman in the chrysalis of *Hyalophora cecropia* (12). AMPs have been found in vertebrates, invertebrates, amphibians, plants, marine organisms, fungi, bacteria, and other organisms (13, 14). AMPs are mainly divided into four categories according to the source as follows: animal-derived AMPs, plant-derived AMPs, microbial-derived AMPs, and synthetic AMPs (15, 16). Almost every class of AMPs comprises the cathelicidin family and the defensin family, which are the two largest groups of all AMPs (17). The cathelicidin family has strong antibacterial activities (18, 19), low haemolytic activity, and high cytotoxicity to drug-resistant strains (20, 21).

The cathelicidin family of porcine AMPs includes four categories as follows: PR-39, PG1-5 (protegrin), PF1-2 (prophenin), and porcine myeloid antimicrobial peptides (PMAPs) (22). PR-39 was initially isolated and purified from the porcine small intestine and has a type II poly-L-proline helix. PR-39 is a cationic host defense peptide rich in proline and arginine with a molecular weight of ~4,700 Da (23–25). PGl-5 was originally isolated from porcine leukocytes and is rich in cysteine and arginine with a molecular weight of ~2,000 Da (26, 27), and PGl-5 has β-hairpin structures. PF1-2 was initially isolated from porcine neutrophils and has an extended helical structure rich in proline and phenylalanine, with a molecular weight of ~8,000 Da (28, 29). PMAPs originate from porcine bone marrow cells and are stored in peripheral polymorphonuclear neutrophil granules in the constitutive form of a prepeptide (22, 30–32). PMAPs exhibit high antimicrobial activity and have a stable α-helix structure (33–38). Among natural AMPs, α-helical structures are the most prevalent and are the same as those in the cathelicidin family AMPs. AMPs with α-helical structures generally exhibit broad-spectrum antibacterial activity, including activity against several clinically antibiotic-resistant strains, such as methicillin-resistant *Staphylococcus aureus*, vancomycin-resistant *Enterococcus faecalis*, and multiresistant *Pseudomonas aeruginosa* (39). However, AMPs that lack the α-helical structures of the porcine cathelicidin family, such as PR-39, are bactericidal against several Gram-negative bacteria (*Escherichia coli* and *Salmonella typhimurium*) and Gram-positive bacteria (*Bacillus megatherium* and *Bacillus subtilis*) (40), but *Proteus vulgaris, P*. *aeruginosa*, and *S*. *aureus* are resistant to PR-39 (41). In addition, PMAPs have the same carpet-like antibacterial mechanism. PMAPs carry many positive charges and easily bind to the negatively charged surface of bacterial membranes, and their binding power to eukaryotic cell membranes is weak or non-existence, resulting in a relatively low haemolytic effect (42). PMAPs have similar antibacterial activity with high-efficiency and broad-spectrum (22). Thus, PMAPs are of great importance in the development of stable, safe, and efficient AMPs of the porcine cathelicidin family.

In recent years, our group has performed research on the structural modification and antimicrobial activity of PMAPs. According to the structure and mechanism of PMAPs, many scholars have designed multiple novel modified peptides with increased stability and antimicrobial activity (43–50). This review mainly summarizes the research progress on the structural characteristics and biological activities of PMAPs in recent years.

## Porcine Myeloid Antimicrobial Peptides

**Figure 1:**
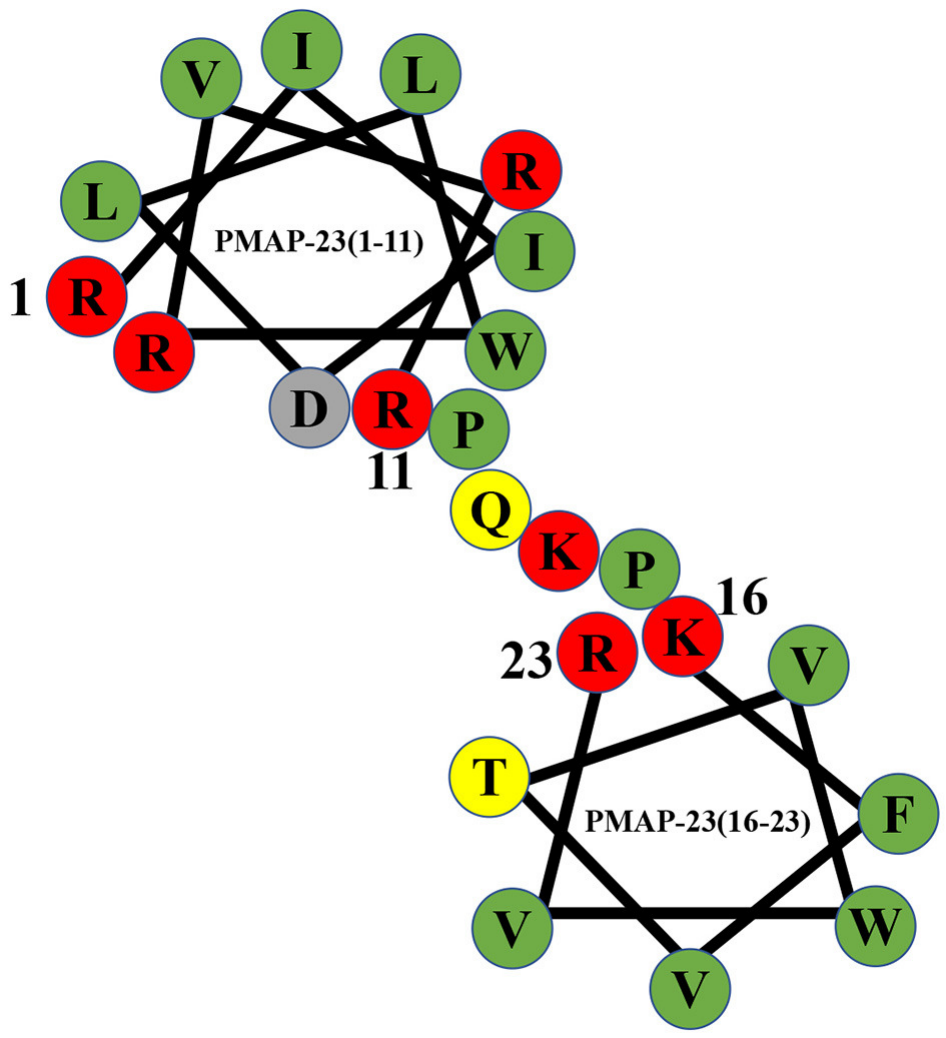
Helical wheel diagram of PMAP-23. Hydrophobic residues are indicated by green circles, and positively charged residues are indicated by red circles. Negatively charged residues are indicated by gray circles, and neutral uncharged residues are indicated by yellow circles. The diagram clearly shows that PMAP-23 has a helix-hinge-helix structure.

**Figure 2:**
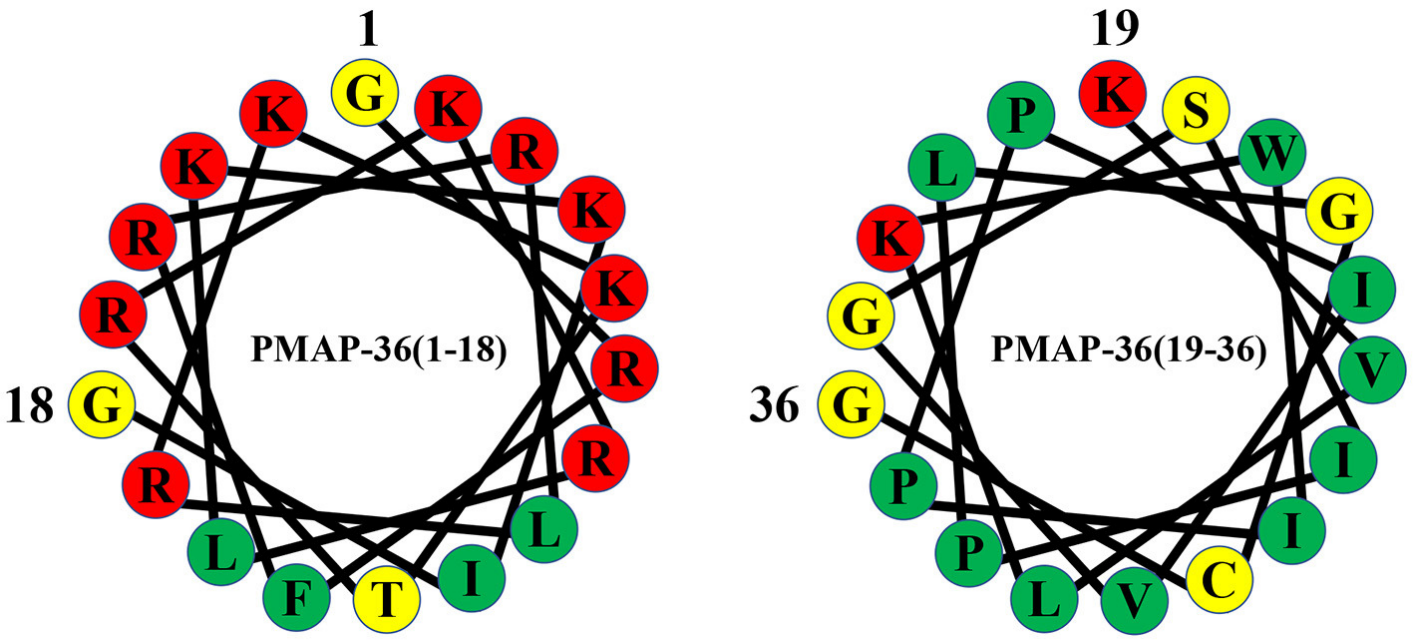
Helical wheel diagram of PMAP-36. Hydrophobic residues are indicated by green circles, and positively charged residues are indicated by red circles. Negatively charged residues are indicated by gray circles, and neutral uncharged residues are indicated by yellow circles.

**Figure 3:**
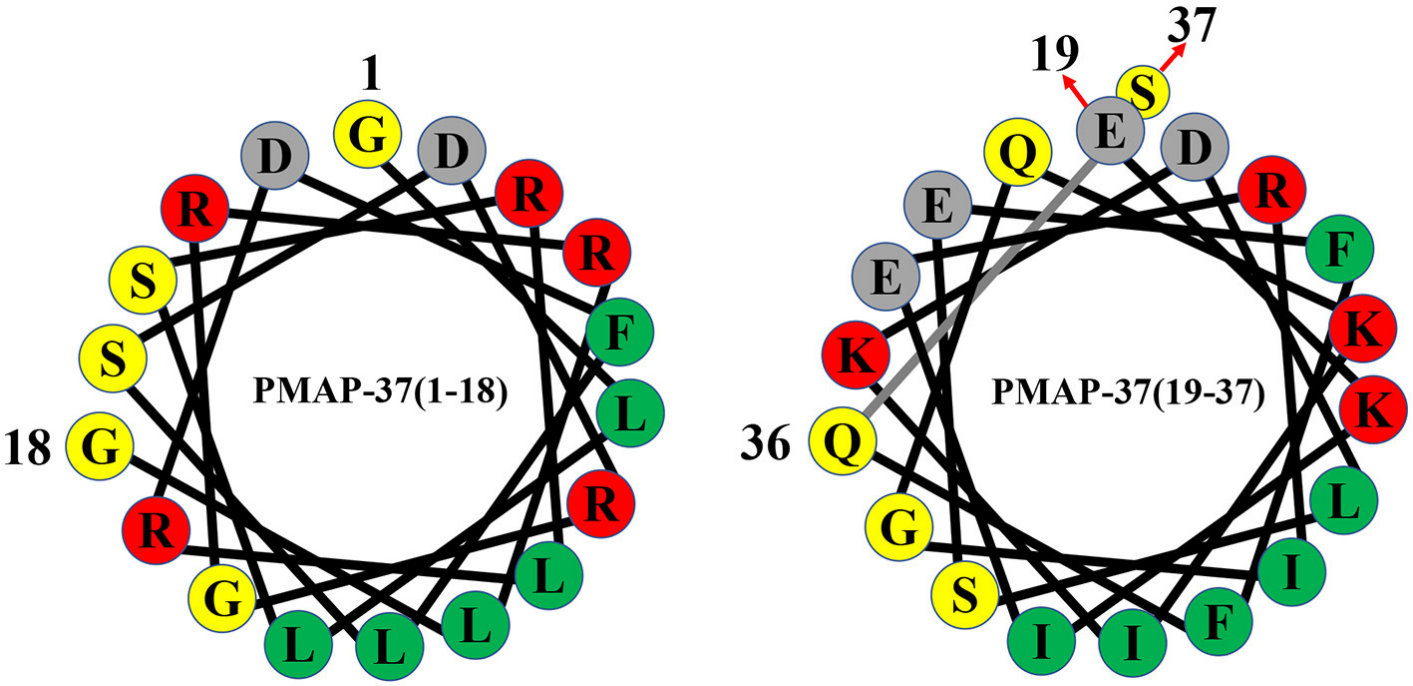
Helical wheel diagram of PMAP-37. Hydrophobic residues are indicated by green circles, and positively charged residues are indicated by red circles. Negatively charged residues are indicated by gray circles, and neutral uncharged residues are indicated by yellow circles.

PMAPs (PMAP-23, PMAP-36, and PMAP-37), a class of cationic AMPs derived from porcine bone marrow cells, are obtained by first extracting RNA from porcine bone marrow cells and then by cDNA cloning, and they can also be obtained by solid-phase synthesis (31). The three peptides are amphiphilic with N-terminal hydrophilicity and C-terminal hydrophobicity, and their sequence similarity is much lower than that of other peptides in the porcine cathelicidin family. PR-39 has seven repeats of Xaa-Pro-Pro-Xaa, and PFs have several repeats of the Phe-Pro-Pro-Pro-Asn-Phe-Pro-Gly-Pro-Arg decamer. Only one to two amino acids differ in PGs, and the other amino acids are the same between them. In addition, the rank order of the peptides is similar (22). The biochemical parameters of PMAPs are shown in Table 1. The helical wheel diagrams of PMAPs are shown in Figures 1–3. The three peptides have an α-helical structure with no toxic side effects to mammalian cells (51). In addition, the α-helix structure, which exists in the secondary structure of various peptides, is an excellent amphiphilic structure with a positively charged hydrophilic side and a negatively charged hydrophobic symmetric side (52, 53).

**Table 1:** Biochemical parameters and structures of PMAPs.

**Peptide**	**Sequence**	**Formula**	**MW^**a**^**	**Charge**	**PI^**b**^**	**GRAVY^**c**^**	**Structure**
PMAP-23	RIIDLLWRVRRPQKPKFVTVWVR	C_140_H_229_N_43_O_28_	2962.63	+7	12.18	−0.296	α-helical
PMAP-36	GRFRRLRKKTRKRLKKIGKVLKWIPPIVGSIPLGCG	C_191_H_336_N_62_O_39_S_1_	4157.22	+13	12.31	−0.461	α-helical
PMAP-37	GLLSRLRDFLSDRGRRLGEKIERIGQKIKDLSEFFQS	C_192_H_320_N_60_O_56_	4365.02	+9	10.24	−0.724	α-helical

a *Molecular weight*;

b *Theoretical isoelectric point*;

c *Grand average of hydropathicity*.

*a, b, c, were calculated by the antimicrobial peptide analysis tool. (https://web.expasy.org/protparam/)*.

PMAP-23 is an α-helical antimicrobial peptide consisting of 23 amino acid residues with a molecular weight of 2962.63 Da, and the amino acid sequence of PMAP-23 is RIIDLLWRVRRPQKPKFVTVWVR (44, 54). PMAP-23 contains seven highly positively charged amino acids (arginines and lysines) and 11 highly hydrophobic residues (55). PMAP-23 also contains two tryptophans with native fluorescence characteristics, and it can be used as a model to observe polypeptide-membrane interactions (56). The peptide possesses a unique helix-hinge-helix structure in the membrane environment, and the α-helix structure is connected by a flexible hinge containing the PXXP motif (35, 57). PMAP-23 has two α-helices in the N-terminal region (Arg1 to Arg10) and the C-terminal region (Phe18 to Arg23) (55). The kink region consisting of two proline residues at positions 12 and 15 increases the amphiphilicity of the charged residues (33). PMAP-23 easily binds to bacterial membranes containing many negatively charged lipids by electrostatic adsorption. Therefore, PMAP-23 has a certain selectivity for the target cell membrane. The bactericidal mechanism of PMAP-23 is a carpet-like mechanism (58). Natural and synthetic PMAP-23 show antibacterial activity against Gram-positive bacteria, Gram-negative bacteria, and fungi (35, 55).

PMAP-36 is a highly cationic amphiphilic α-helical antimicrobial peptide consisting of 36 amino acid residues with a molecular weight of 4157.22 Da (47, 59), and the complete amino acid sequence of PMAP-36 is GRFRRLRKKTRKRLKKIGKVLKWIPPIVGSIPLGCG (59). PMAP-36 has the highest net charge with 13 positively charged amino acids (seven lysines and six arginines). Cationic amino acids are mostly in the N-terminus and form an α-helical structure at the N-terminus. The high net charge contributes to the electrostatic interaction between PMAP-36 and negatively charged molecules on the surface of bacterial cell membranes (60, 61). The peptide also contains 21 hydrophobic amino acids in the C-terminus (34). The highly hydrophobic tail plays an important role in broad-spectrum antimicrobial activity (61, 62). The C-terminal 35th amino acid of PMAP-36 is a cysteine, which can dimerize two PMAP-36 peptides through intermolecular disulphide bonds, and these peptides are stored in leukocytes as dimers (62). Although monomers and dimers show rapid and effective bactericidal activity against most microorganisms, dimeric peptides have a more stable conformation, longer bacteriostatic time, and lower medium sensitivity (34, 62).

PMAP-37 is an amphiphilic antimicrobial peptide that contains 37 amino acid residues with a molecular weight of 4365.02 Da (38, 50), and the amino acid sequence of PMAP-37 is GLLSRLRDFLSDRGRRLGEKIERIGQKIKDLSEFFQS (48, 49). PMAP-37 has the highest hydrophobicity and the lowest net charge (22). In trifluoroethanol, PMAP-37 can form an α-helix-dominated structure from an irregular coiled structure, and it is a typical amphiphilic α-helix cationic antimicrobial peptide (38). Moreover, PMAP-37 is the strongest membrane-active peptide, which can permeate the intracellular membrane of bacteria at 0.2-1 μM (37, 63). The 15-32 stretch of PMAP-37 is significantly similar to the N-terminal extension of cecropin B and A in *Drosophila melanogaster* and *Cecropia hyalophora*, respectively (38). Therefore, PMAP-37 provides a unique example of a sequence convergence structure for the scientific community.

## Biological Activity of PMAPs

### Antibacterial Activity

#### Antibacterial Mechanism of PMAPs

**Figure 4:**
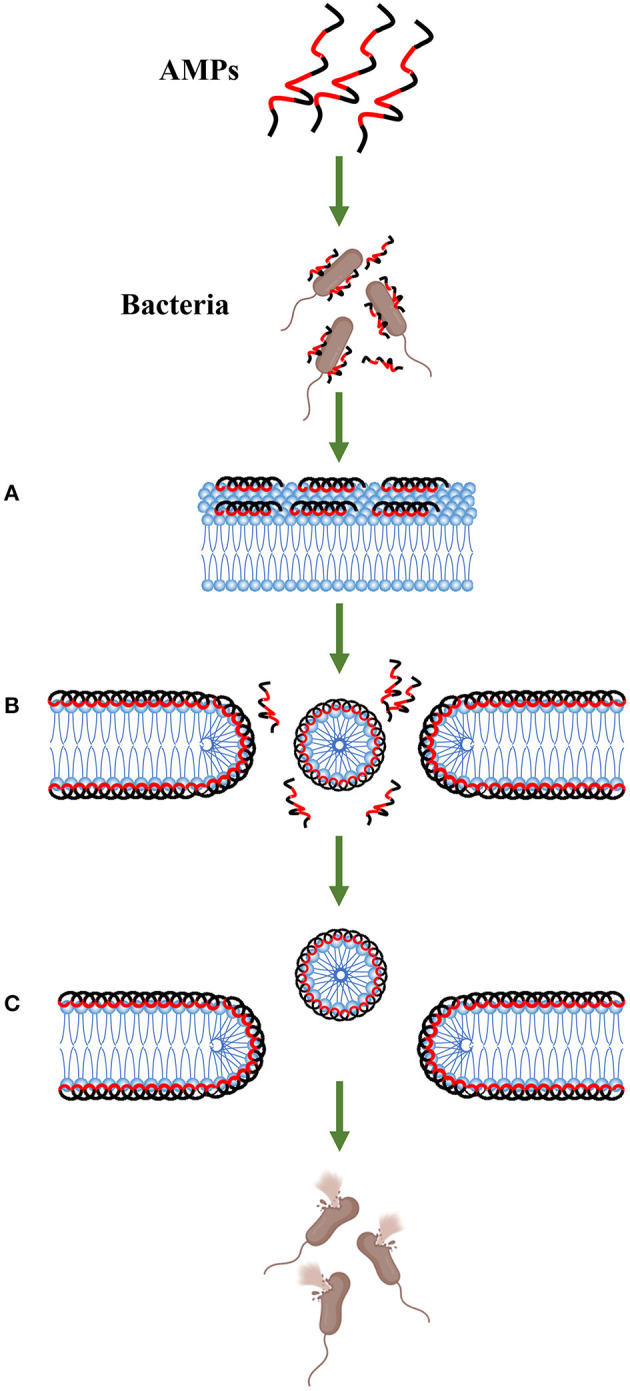
Carpet-like mechanism. Antimicrobial peptides aggregate on the surface of the bacteria and remain in equilibrium with the membrane through electrostatic interactions. The hydrophobic surface (red area) faces the membrane, and the hydrophilic surface (black area) faces the solvent (step A). When the peptide monomer reaches a threshold concentration, the membrane is permeated, and a temporary pore is formed (step B). This process may also cause the membrane to disintegrate into micelles (step C).

AMPs can interact with the cell membrane of bacteria and then destroy the integrity of the membrane, ultimately leading to leakage of the cell contents and killing the cells (13, 53). Various models for the interaction of AMPs with cell membranes have been proposed, such as barrel-stave, carpet-like, and toroidal-pore models (13, 35, 64), and the methods of damaging the membrane are different in each model. For example, in the barrel-stave model, AMPs insert into the cell membrane phospholipid bilayer from a direction perpendicular to the cell membrane as multimers by polymerizing with the phospholipid bilayer, thereby forming ion channels across the cell membrane that rupture the membrane. In the carpet-like model, AMPs saturate the membrane and then disrupt the cell membrane (64). Here, we mainly introduce the mechanism of the carpet-like model of PMAPs (38, 58, 61, 65) (Figure 4). AMP molecules aggregate on the surface of the membrane in large quantities and remain in equilibrium with the membrane through electrostatic interactions, forming a carpet-like morphology (66). The peptide then begins to reorient. The hydrophobic region of the peptide is oriented preferentially toward and bound to the phospholipid head groups of the membrane, and its hydrophilic region is oriented toward the solvent. Because the peptide consistently makes contacts with the phospholipid head during the action, there is no need to insert into the hydrophobic core of the membrane. When the peptide reaches a threshold concentration, the membrane fluidity changes until the membrane structure is unstable. The peptide penetrates the membrane, or the membrane disintegrates into micelles, allowing the cellular contents to extravasate and ultimately leading to bacterial death (67–69). Because transmembrane pores may also form when the concentration of some peptides is below a threshold, the mechanism by which AMPs disrupt the membrane has an important relationship with the concentration (13, 35, 64). PMAPs increase the contents of NO and reactive oxygen species (ROS) in *E*. *coli* cells, causing DNA strand oxidative breaks and cell death. In addition, DNA damage is also part of the bacterial death mechanism (70).

#### Antibacterial Activity of PMAP-23

PMAP-23 has high antimicrobial activity against Gram-negative and Gram-positive bacteria (71). Compared to the antimicrobial activity of the conventional ampicillin antibiotic, the antimicrobial activities of PMAP-23 and PMAP-23N (C-terminal amidated) against Gram-negative bacteria are 10- to 80-fold higher (72). However, the MIC of PMAP-23 against *S*. *aureus* is slightly higher than that against other bacteria (56). The N-terminus of PMAP-23 is significantly associated with its antimicrobial activity, but salt tolerance is dependent on the C-terminal amino acids (33). NaCl has an inhibitory effect on the activity of the truncated peptide of PMAP-23 (1–17, 73), which may be due to the reduction in positively charged amino acids and the consequent decrease in electrostatic interactions between the polypeptide and the bacterial membrane (74), and the stability of the α-helix structure is also essential for antibacterial activity (75). The reduced activity of truncated peptides may be related to the disruption of the α-helix structure. In the C-terminal truncated peptide of PMAP (1–13, 73), the antimicrobial activity is enhanced after replacing the negatively charged aspartic acid with a positively charged lysine, which may be explained by the fact that increasing the net charge of the polypeptide enhances the antibacterial activity (76). Indeed, the principle applies equally to helical AMPs of other cathelicidin families. For example, cathelicidin-BF (Cath-BF) is a helical AMP extracted from *Bungarus fasciatus* and contains 34 amino acid residues. Cath-A and Cath-B, two analogs of Cath-BF, have positively charged lysine substitutions that increase the net charge by three and two, respectively. Among them, Cath-A exhibits 2-fold greater antibacterial activity against methicillin-resistant *S. aureus* than the parent Cath-BF peptide. For *E. coli* ATCC 25922, although the antibacterial activity of Cath-A is similar to that of Cath-BF, the activity of Cath-B is enhanced by 4-fold (77). CRAMP-18 is an AMP derived from the murine cathelicidin family, and the K^2^-CRAMP-18 derivative peptide has an increased net charge. The antimicrobial activity of K^2^-CRAMP-18 against Gram-negative bacteria (such as *E*. *coli* and *S*. *Typhimurium*), Gram-positive bacteria (such as *S*. *pyogenes* and *S*. *aureus*), and fungi (such as *Albicans*) is increased by 8- to 16-fold with almost no haemolytic activity. Therefore, the potential antibacterial activity of K^2^-CRAMP-18 is enhanced by increasing the positive net charge of AMPs (78).

The fifth amino acid (leucine) of the PMAP-23-derived peptides, PMAP-23R (Leu5-Arg), PMAP-23I (Thr19-Ile), and PMAP-23RI (Leu5-Arg and Thr19-Ile), is substituted with an arginine to increase the net charge, and the nineteenth amino acid (threonine) of PMAP-23 is substituted with an isoleucine to increase the hydrophobicity (43). Both the antimicrobial spectrum and activity of PMAP-23 are significantly increased, especially against Gram-negative bacteria. PMAP-23R has a higher antimicrobial activity than PMAP-23, which may be due to the increase in positive charge, allowing PMAP-23R to more easily bind to negatively charged bacterial membranes (79). The increased antimicrobial activity of PMAP-23I is perhaps due to the increased hydrophobicity, which in turn also increases the affinity of PMAP-23I for membranes, allowing it to be more easily entrapped in the phospholipid layer of bacteria (80, 81). PMAP-23RI has an increased net charge and hydrophobicity, resulting in the best antibacterial effect. Similar to PMAP-23, human LL-37 is an α-helical AMP from the cathelicidin family. Nagaoka utilized the amphipathic 18-mer peptide (K^15^-V^32^) of LL-37 as a template and designed a new peptide, 18-mer LL, by enhancing its hydrophobicity using two L residues to replace E^16^ and K^25^ followed by the replacement of Q^22^, D^26^, and N^30^ of the 18-mer LL with three K residues to enhance its positive charge to create the derivative peptide, 18-mer LLKKK. Compared with the parental 18-mer peptide, 18-mer LL has enhanced hydrophobicity and better antibacterial efficacy against both Gram-positive (such as MRSA and *S. pyogenes*) and Gram-negative bacteria (such as *E. coli* and *P. aeruginosa*); however, the 18-mer LLKKK peptide, which has both increased hydrophobicity and positive charge, exhibits the best antibacterial efficacy (82). Therefore, the antimicrobial activity of AMPs is enhanced by increasing their hydrophobicity and net charge. Similarly, fatty acid modification with hydrophobicity can also serve as a strategy to improve antibacterial activity. Fatty acid modification of a certain chain length (carbon number 4-14) enhances the hydrophobicity of the polypeptide, which enhances the antimicrobial activity (83). PMAP-23RI-Dec was designed by modifying the C-terminus of PMAP-23RI with decanoic acid. The antibacterial activities of PMAP-23RI-Dec are 4-fold higher than those of PMAP-23RI (except against *P*. *aeruginosa*). In addition, mouse models of wound infection or an abscess caused by *S. aureus* and *P. aeruginosa* bacteria have been treated by subcutaneous injection of AMPs or antibiotics into mice, respectively, demonstrated that PMAP-23RI-Dec is more effective in treating infected mice (44).

#### Antibacterial Activity of PMAP-36

PMAP-36 is an effective antimicrobial peptide against both Gram-positive and Gram-negative bacteria (34). In general, *S*. *typhimurium* is susceptible to most AMPs, but PMAP-36 has relatively low activity. However, PMAP-36 has high antimicrobial activity against *P. aeruginosa, Bacillus megaterium*, and *S. aureus* (84). In addition, PMAP-36 activity has been compared to the antibacterial activity of two widely studied polypeptides, human LL-37 and chicken Cath-2. PMAP-36 shows the strongest antimicrobial activity against *E. coli* O78 and has a significant inhibitory effect on *E. coli* O78 at 2.5 μM (60).

The 24 residue truncated peptide, GI24, which contains all cationic amino acids of PMAP-36, has antibacterial activity consistent with that of PMAP-36 (61), demonstrated that the antibacterial activity of PMAP-36 is closely related to the positively charged N-terminal amino acids. The RI21 and RI18 truncated peptides of PMAP-36 also show good antibacterial activity against bacteria with higher antibacterial activity than melittin and significantly lower haemolytic activity than PMAP-36 and melittin. In addition, both PMAP-36 and its derived peptides have potent antimicrobial activity against *B*. *subtilis* (36). Therefore, reducing the chain length of PMAP-36 within a certain range not only maintains antimicrobial activity but also reduces haemolysis.

PMAP-36 shows antimicrobial activity against both Gram-negative and Gram-positive bacteria, and it is 4- to 128-fold more effective than gentamicin and streptomycin. In addition, antibiotic-peptide combinations have good antimicrobial activity against *E. coli* ATCC 25922 and *S. aureus* ATCC 29213. When PMAP-36 is combined with gentamicin, it has a synergistic effect on *E. coli* and a partial synergistic effect on *S. aureus* (59). Therefore, the synergistic antimicrobial activity of the peptide and antibiotics is a good prospective application.

PMAP-36PW and PMAP-36PK are two new peptides designed by replacing the prolines at positions 25 and 26 of PMAP-36 with tryptophan and lysine, which increases the hydrophobicity and positive charge as well as extends the α-helical structure of PMAP-36. PMAP-36 has no bacteriostatic effect on *E. coli* K88, while PMAP-36PW and PMAP-36PK have an antibacterial effect on *E. coli* K88. Compared to that of PMAP-36, the antibacterial activities of PMAP-36PW and PMAP-36PK against Gram-positive bacteria and Gram-negative bacteria are also significantly increased, especially against *Listeria monocytogenes*, with increases in antibacterial activity of 4- to 8-fold. In addition, in murine animal model experiments of acute infection with *Salmonella choleraesuis* and *L. monocytogenes*, the modified peptide shows therapeutic efficacy comparable to that of the ceftiofur sodium antibiotic (45). Myristic acid has been used to modify the N-terminus of PMAP-36PW to improve hydrophobicity, resulting in a novel modified peptide, Myr-36PW. Compared with PMAP-36PW, Myr-36PW has a significantly lower MIC against Gram-negative bacteria and Gram-positive bacteria, especially *L. monocytogenes* CICC 21634. The MIC of Myr-36PW is reduced by 4-fold, and it has effective antibiofilm activity. In addition, a previous study investigating the effects of Myr-36PW in mice infected with *S. aureus* ATCC 25923 and *P. aeruginosa* GIM1.551, Myr-36PW showed a prominent therapeutic effect on mouse pneumonia and peritonitis experiments compared with those of PMAP-36PW, and it promoted abscess reduction and wound healing in infected mice. Compared to the penicillin antibiotic, there was no significant difference in the therapeutic effect (47). This study provides an important reference value for the development of novel antimicrobial drugs.

#### Antibacterial Activity of PMAP-37

PMAP-37 has potent antimicrobial activity against both Gram-positive (such as *B. megaterium* and *S. aureus*) and especially Gram-negative bacteria (such as *E. coli, S. typhimurium*, and *P. aeruginosa*) (38). Generally, the antimicrobial activity of AMPs is enhanced by modifying the primary and secondary structures of AMPs in a certain range to increase the net charge of AMPs, prolong their α-helix, and increase their hydrophobicity (85–88). PMAP-37 (R13-I) and PMAP-37 (K20/27-I) are two analogs with substitutions of isoleucine for arginine at position 13 and isoleucine for lysine at positions 20 and 27, resulting in increased hydrophobicity of PMAP-37. The sensitivity of Gram-negative bacteria to PMAP-37 and its analogs is higher than that of Gram-positive bacteria, and the MIC of PMAP-37 (R13-I) for Gram-negative bacteria is lower than that of PMAP-37. Moreover, PMAP-37 (K20/27-I) has antimicrobial activity against *Shigella flexneri* CICC 21534, especially *S. typhimurium* SL1344, with ~4-fold increases in antimicrobial activity, and it has obvious therapeutic effects on mice infected with *S. aureus* ATCC 25923 and *S. typhimurium* SL1344 (48). The antimicrobial activity can be enhanced by increasing the net charge of the polypeptide to a certain range (89). Three PMAP-37 analogs, PMAP-37 (F9-R), PMAP-37 (F34-R), and PMAP-37 (F9/34-R), were designed by replacing the phenylalanine at positions 9 and 34 of PMAP-37 with arginine. In addition to the bacteriostatic activity against *S. flexneri* CICC 21534, PMAP-37 has higher bacteriostatic activity against other Gram-negative bacteria tested. Compared to PMAP-37, PMAP-37 (F9-R) and PMAP-37 (F9/34-R) have stronger antimicrobial activity against *S. flexneri* CICC 21534, and PMAP-37 (F34-R) has the highest antimicrobial activity (49).

A novel AMP, Chol-37 (F34-R), was designed by modifying the N-terminus of PMAP-37 (F34-R) with cholesterol, which increases the hydrophobicity of the peptide. The MICs of the modified Chol-37 (F34-R) peptide against *S. aureus* ATCC 25923 and *S. typhimurium* SL1344 are 2-fold lower than those against PMAP-37 (F34-R) and 4-fold lower than those of *P. aeruginosa* GIM1.551 and *L. monocytogenes* CICC 21634, and Chol-37 (F34-R) exhibits effective antibiofilm activity, low toxicity, and no haemolytic activity. In addition, in the model of knife injury and abscess, Chol-37 (F34-R) shows better wound healing and abscess reduction as well as a stronger ability to remove bacteria. In the peritonitis model infected with *S. aureus* ATCC 25923, the *in vivo* treatment and pathological injury recovery of Chol-37 (F34-R) are similar to those of benzylpenicillin potassium, surpassing ampicillin sodium; Chol-37 (F34-R) also shows strong antimicrobial activity, suggesting good application prospects (50).

Similar to PMAP-23 and PMAP-36, PMAP-37 exhibits a broad antimicrobial spectrum with MICs of ~1–4 μM. In addition, PMAP-37 also has haemolytic activity, and it has a haemolytic effect on human red blood cells at 10–15 μM (38). However, PMAP-23 and PMAP-36 do not lyse erythrocytes at concentrations higher than 100 μM (31, 38). At present, there are relatively few studies on PMAP-37.

### Antifungal Activity

#### Antifungal Activity of PMAP-23

The antifungal mechanism of AMPs results in the loss of fungal cell activity mainly caused by the formation of transmembrane channels, which increases membrane permeability, leading to the destruction of microbial cell structure (90, 91). Kim studied the antifungal mechanism of PMAP-23 against *Candida albicans* at low concentrations, demonstrating that the concentration of PMAP-23 that causes bacterial membrane depolarization and potassium ion efflux (2.5 μM) does not disrupt the cell membrane. Lower concentrations of PMAP-23 result in higher levels of calcium ions in the cytoplasm and mitochondria, which causes *C. albicans* apoptosis. Mitochondrial calcium-induced ROS are the main factors triggering apoptosis. Therefore, the antifungal mechanism of PMAP-23 not only destroys biofilms but also induces apoptosis (35).

PMAP-23 has significant antifungal activity against *Saccharomyces cerevisiae* KCTC 7296, *Trichosporon beigelii* KCTC 7707, and *C. albicans* TIMM 1768. In particular, PMAP-23 has the highest antifungal activity against *T. beigelii* KCTC 7707, higher antifungal activity against *C. albicans* TIMM 1768, and no haemolytic activity. In addition, PMAP-23 inhibits the apical growth of *Trichophyton rubrum*, which has an obvious antigrowth effect (92).

Based on substitution of tryptophan for amino acids 10, 13, or 14 of PMAP-23, several PMAP-23 analogs have been designed, resulting in enhanced hydrophobicity of the parent peptide. Among these, the analogous P6 peptide has tryptophan at positions 10, 13, and 14 of PMAP-23 and has strong fungicidal activity. Compared to PMAP-23 and other analogs, P6 has the strongest antifungal effect on *C. albicans*, and the MIC of P6 is decreased by 4- to 8-fold compared to that of PMAP-23. P6 destroys the hyphal morphology of *C. albicans* by destroying the cell membrane of fungi, thereby resulting in a fungicidal effect on spores and changing the morphology of *C. albicans*. Therefore, fungicidal activity also significantly increases by enhancing the hydrophobicity of PMAP-23 (93).

#### Antifungal Activity of PMAP-36

Both monomers and dimers of PMAP-36 have antifungal activity against *C. albicans*, while PMAP-36 (1–18, 73), a C-terminal truncated peptide of PMAP-36, has no antifungal activity against *C. albicans*. Thus, the C-terminus is important for PMAP-36 to exert antifungal activity (34). A series of truncated peptides of PMAP-36 have been designed by amino acid truncation, and the antifungal activity and cell selectivity of these peptides against *Candida* have been investigated and compared with those of melittin (36). RI18 has high antifungal activity and the highest selectivity index for fungi with a value 108-fold higher than that of PMAP-36. RI21 and RI18 have high antifungal activity against *C. albicans* with MICs ~16–21-fold higher than that of PMAP-36, and the haemolytic activities are lower than those of PMAP-36 and melittin. Of these, RI21 has the lowest MIC against Candida with a value ~6-fold lower than that of PMAP-36. In addition, all the peptides showed strong antifungal activity against *Candida tropicalis* 2.1975 comparable to the antifungal activity of melittin (36), indicating that reducing the chain length of PMAP-36 not only maintains high antimicrobial activity but also reduces haemolysis.

### Antiparasitic Activity

PMAP-23 not only has an inhibitory effect on bacteria and fungi but also has an inhibitory effect on the eggs and worms of Caenorhabditis elegans (94). *C*. *elegans* cells treated with PMAP-23 and stained with propidium iodide have significantly higher fluorescence intensity than normal cells. Confocal microscopy has also confirmed that PMAP-23 penetrates the cell membrane and is present in the eggshell and plasma membrane of *C*. *elegans*. After 20 h of incubation, PMAP-23 is inactive on the eggs but active on the worms. When incubated for 60 h, PMAP-23 shows some activity on the eggs. This result may be due to the presence of the shell layer in the eggs, which affects the hatching rate. When the concentration of PMAP-23 is 200 μM, the hatchability of worm eggs does not decrease, but when the concentration is 25 μM, the mobility of worms becomes highly sensitive (94, 95). Therefore, PMAP-23 may exert its anti-worm nodule role by destroying the cell membrane structure of *C*. *elegans*.

### Antitumour Activity

PMAP-23 shows some antitumour activity against both Jurkat and SNU 601 tumor cells, whereas P6 shows the highest antitumour activity with an activity ~4-fold that of PMAP-23, confirming that increased hydrophobicity of PMAP-23 contributes to the antitumour activity (93). Shin SY designed two modified peptides, PMAP1 and PMAP2, by replacing proline at positions 12 and 15 of PMAP-23 with alanine, respectively, and investigated the antitumour activity of PMAP-23 and its engineered peptides in tumor cells (human chronic myeloid leukemia K-562 cells, human acute T-cell leukemia Jurkat cells, human lung cancer A-549 cells, and human breast cancer MDA-MB-361 cells). The antitumour activity of PMAP-23 is 2-fold higher than that of PMAP1, while PMAP2 has no inhibitory effect on all tumor cells, indicating that the two prolines in PMAP-23 play an important role in inhibiting tumor cells. In addition, the 15th proline in PMAP-23 is more important than the 12th proline in the antitumour effect, and PMAP-23 does not lyse human erythrocytes even at 100 μM (96). The biological activities of PMAPs are summarized in Table 2.

**Table 2:** Biological activity of PMAPs.

**Peptide**	**Biological activity**	**Strains/parasites/tumor cells**	**References**
PMAP-23	Antibacterial	*E. coli, S. typhimurium, P. aeruginosa, B. megaterium, S. aureus, B. subtilis, Streptococcus pyogenes, Bacillus globigii, P*. *vulgaris, Staphylococcus epidermidis, L. monocytogenes, S. choleraesuis, Pasteurella multocida, S. flexneri, Enterococcus faecalis*	(33, 43, 44, 56, 71, 72, 76, 79)
	Antifungal	*C. albicans, T. beigelii, S. cerevisiae*	(35, 92, 93)
	Antiparasitic	*C*. *elegans*	(94, 95)
	Antitumour	*SNU601, MDA-MB-361, Jurkat, A-549, K-562*	(93, 96)
PMAP-36	Antibacterial	*E. coli, S. typhimurium, P. aeruginosa, B. megaterium, S. aureus, B. subtilis, S. epidermidis, L. monocytogenes, S. choleraesuis, MRSA, Pasteurella avium, Salmonella enteritidis*	(34, 36, 45, 47, 59–61, 84)
	Antifungal	*C. albicans, Cryptococcus neoformans, C. tropicalis, Candida krusei*	(34, 36)
PMAP-37	Antibacterial	*E. coli, S. typhimurium, P. aeruginosa, B. megaterium, S. aureus, B. subtilis, S. epidermidis, L. monocytogenes, S. choleraesuis, P. avium*	(38, 48–50)

## Conclusion and Future Direction

In the past 80 years, ~5,000 AMPs have been discovered or synthesized, and some of these are in preclinical and clinical development. Compared with antibiotics, AMPs have many advantages, such as broad antimicrobial spectrum, efficacy, selectivity, high potency, low toxicity, low accumulation in tissues, and difficulty producing drug resistance (7, 73). PMAPs have high antimicrobial activity against bacteria and fungi (35, 36, 38, 71, 84), and they have certain inhibitory effects on tumors and parasites (94–96). At present, scholars worldwide have designed several new modified peptides through the modification of primary structure and secondary structure. Compared with the original peptides, the modified peptides have higher antimicrobial activity, better therapeutic effect, and lower toxicity. PAMPs with high efficacy and broad antimicrobial spectrum are unlikely to develop drug resistance in the future, thereby presenting new options in terms of developing alternatives to antibiotics and clinical treatment. In addition, PMAP genes may be introduced into animals to confer specific disease resistance by genetic engineering techniques. PMAPs can be applied in animal husbandry as feed additives, resulting in less reliance on traditional antibiotics. However, the antibacterial activity of natural PMAPs is not ideal as PMAPs are easily degraded *in vivo*. Importantly, the mechanism of how PMAPs bind to intracellular targets and inhibit cell wall, DNA, RNA, and protein synthesis is not well-understood. Further studies on the relationship between structure and activity as well as the mechanism of action are needed to provide an adequate theoretical basis for the design of safe, stable, and efficient AMPs. Many drugs in clinical trials have some type of chemical modification to improve their druggability to improve the possibility of clinical application (7).

Among the structural optimization strategies for PMAPs, amino acid modification and fatty acid modification have been the most effective ways to improve their biological activity. In addition, the application of nanotechnology to modify AMPs or the use of hydrogel-encapsulated AMPs to make hydrogel preparations and the production of controlled drug release systems are also effective modification strategies (97, 98). Currently, studies of PMAPs using these two techniques have not been reported. Therefore, nanomodification and hydrogel preparation may be a new research direction for PMAPs in the future. In summary, continuous research on structural modifications of PMAPs and their mechanisms of action will help to limit the impact of antimicrobial resistance and reduce concerns about the public health hazards caused by antimicrobial resistance.

## Author Contributions

SS and TS conducted the literature review and wrote the manuscript. YL and LC revised the manuscript. CW and CL revised the manuscript and administrated the project. All authors read and approved the final manuscript.

## Conflict of Interest

The authors declare that the research was conducted in the absence of any commercial or financial relationships that could be construed as a potential conflict of interest.
